# Re-hospitalizations within 30-days and mortality outcomes among severely visually impaired and blind patients: analysis of the National Readmission Database

**DOI:** 10.1186/s12886-023-03051-8

**Published:** 2023-08-08

**Authors:** Michael Fatuyi, Oladipupo Anibire, Che Matthew Harris

**Affiliations:** 1 Department of Medicine, TriHealth Good Samaritan Hospital Program, Cincinnati, OH United States of America; 2 Meharry Medical College, Nashville, TN United States of America; 3 Department of Medicine, Johns Hopkins School of Medicine, Johns Hopkins Bayview Medical Center, Baltimore, MD United States of America

**Keywords:** Hospitalization, Vision impairment, Blindness, Readmissions, Outcomes

## Abstract

**Background:**

Readmissions and in-hospital mortality among patients with severe vision impairment or blindness (SVI/B) has not been fully studied. We investigated hospital outcomes for adults with SVI/B in the United States.

**Methods:**

Using the Nationwide Readmission Database year 2017, we analyzed primary outcomes for thirty-day readmission rates for patients with and without SVI/B. Secondary outcomes were in-hospital mortality rates for readmitted patients, in-hospital mortality rates for index patients, the five most common principal diagnoses for readmission, and resource utilization.

**Results:**

34,558 patients had an index admission for SVI/B vs. 24,600,000 who did not. Patients with SVI/B had a 13.3% [4,383] readmission rate within 30 days compared to 8.4% [2,033,329] without SVI/B. Compared to readmitted patients without SVI/B patients, those with SVI/B were older (mean [SD] age: 64.4 [SD ± 19] vs. 61.4 [SD ± 20] years) and had more comorbidities (Charlson comorbidity score ≥ 3: 79.2% [ 3,471] vs. 60.9% [1,238,299]). The mortality rate among patients readmitted with SVI/B was 5.38% [236] vs. 4.02% [81,740] for patients without SVI/B, p-value = 0.016. Top reasons for readmissions among patients with SVI/B included sepsis 12% [526], heart failure 10.5% [460)], acute renal failure 4.4% [193], complications due to type II diabetes mellitus 4.1% [178], and pneumonia 2.7% [118]. The mean length of stay for readmitted patients with SVI/B was 6.3 days (confidence interval [CI]: 6.0-6.7 days), vs. 5.6 days for patients without SVI/B (CI: 5.5–5.8 days), p-value < 0.01. The mean hospital charges for readmitted patients with SVI/B was $57,202 (CI: $53,712–$61,292) vs. $51,582 (CI: $49,966–$53,198), p-value < 0.01.

**Conclusion:**

Patients with SVI/B had higher readmission rates and greater mortality on readmissions than those without SVI/B. Interventional studies for optimal discharge strategies are critically needed to improve clinical and resource utilization outcomes in patients with SVI/B.

**Supplementary Information:**

The online version contains supplementary material available at 10.1186/s12886-023-03051-8.

## Background

Severe vision impairment or blindness (SVI/B) can have devastating consequences on affected individuals’ quality of life and health. By 2050, over 8 million Americans are anticipated to develop SVI/B [1]. In addition, yearly total healthcare expenditures needed to address visual problems in the United States have been estimated to be billions of dollars [2, 3]. Research has shown that hospitalizations among patients with SVI/B are associated with high inpatient healthcare costs and hospital readmissions in older adults [4]. However, the study of readmissions with greater specificity through the International Classification of Diseases, 10th Revision, Clinical Modification (ICD-10) codes has not been carried out. In addition, hospitalizations of patients with SVI/B have been associated with increased in-hospital mortality for adults eighteen years of age and older [5]. Causes for higher mortality in the SVI/B population are unclear, though medical errors or patient-provider miscommunication could be factors [4]. Given these realities, some facilities are beginning to implement education programs to help hospital teams improve care for these patients [6]. For example, a quality improvement project conducted at one hospital sought to determine the needs of hospitalized patients with vision impairment and the means to increase staff education. Staff education was conducted through online training modules, and the development of hospital guidelines on how to care for visually impaired patients was established [6]. The project successfully identified patient responses on what would make them feel safer in the hospital, such as alert bracelets. Screening for vision impairment during hospitalization may offer another opportunity to identify patients with the condition and thus improve their hospital course. Hospital care teams can only intervene and implement care for patients who are known to be visually impaired, which may not always be apparent. A study by Press and colleagues displayed the importance of vision in hospitalized patients’ care. Their study showed that providing low-cost, non-prescription “readers” to patients corrected over 80% of patient vision impairment [7]. The quality of care in the hospital also should be expected to improve as a result. As Press explained, being able to read consent forms for procedures is essential for patient safety and helps patients better understand their diagnostic and management plans. Furthermore, screening patients for vision impairment and correcting the issue during hospitalization may reduce the risk of delirium, which is also associated with poor in-hospital patient outcomes [7].

Unfortunately, screening has not been widely implemented nationally in the United States. Though these studies are some of the first steps toward improving the care of visually impaired patients, most U.S. hospitals may lack the resources necessary to adequately address the care for this patient population [4]. This places patients with SVI/B at high risk for poor hospital and clinical outcomes. Using the Nationwide Readmission Database (NRD) for the year 2017, we hypothesized that patients with SVI/B would have greater readmissions, higher in-hospital mortality, and greater healthcare utilization, suggested by hospital charges and length of stay, than patients without SVI/B.

## Methods

### Data source

This is a retrospective cohort study using the Healthcare Cost and Utilization Project’s Nationwide Readmission Database (NRD) for the year 2017. The NRD is the largest publicly available all-payer inpatient healthcare readmission database in the United States. For the year 2017, unweighted, the database approximates 17,978,754 discharges from 28 states [8]. Weighted, the database estimates 35,790,513 discharges from 2,454 participating hospitals. It is designed as a stratified probability sample to represent all nonfederal acute care inpatient hospitalizations nationwide. Briefly, hospitals are stratified according to ownership/control, the number of beds, teaching status, urban/rural location, and geographic region. A 20% probability sample of all hospitals within each stratum is then collected. Those hospital discharges are recorded, and information about patients’ demographics, principal and secondary diagnoses, vital status at discharge, readmission, and resource use, including length of stay, procedures performed, and total hospitalization costs and charges, are entered into the NRD.

To make the NRD nationally representative, individual discharge is weighted (total number of discharges from all acute care hospitals in the United States divided by the number of discharges included in the 20% sampling). The NRD contains both hospital-level and patient-level data. Up to forty discharge diagnoses and twenty-five procedures data are collected for an individual patient using the International Classification of Diseases, Tenth Revision, Clinical Modification (ICD10-CM).

### Study population

Our study population included patients aged ≥ 18 years. We selected patients with severe bilateral visual impairment or blindness as described by ICD-10 CM codes (eye category 2 through 5 for either eye) [9]. Supplementary material provides ICD-10 codes for specific descriptions of categories for each level of SVI/B. Severe vision impairment has been defined as individuals with visual acuity worse than 6/60, and blindness as those with visual acuity worse than 3/60 [9, 10]. Because the NRD captures admission purely on a calendar year basis (i.e., January 1 through December 31) without a link to the previous or following year, index hospitalization discharges occurring in December were also excluded [8]. The institutional review board of TriHealth Good Samaritan Hospital deemed the research project exempt from approval because it is a retrospective review of de-identified data.

### Study outcomes

All patients in NRD are assigned unique database identification numbers that can be used to identify each patient’s admissions within the state during the calendar year 2017. The primary outcome of this study was hospital readmission. Readmission was defined as any admission for any principal diagnosis within 30 days of the index admission. If patients had multiple readmissions within 30 days of discharge, only the first readmission was counted. Same-day admissions and discharges were excluded. Patients admitted for nonspecific traumatic diagnoses were excluded using the NECODE. The NECODE provides a method of classifying injuries. The NECODE used for nonspecific traumatic readmission exclusion were ICD-10 codes which are “S, T, V, and Y.” These codes do not suggest that the index hospitalization contributed in any way to hospital readmissions. Each code is represented as follows: ICD-10 code S: injuries to the head, neck, thorax, abdomen, lower back, lumbar spine, pelvis, and external genitals, which do not specify if the injury is due to a motor vehicle accident, external force like a knife, or other occupational hazards. ICD-10 code T: burns and corrosions of the external body surface, frostbite, and poisoning by the adverse effect of and underdosing of drugs, medicaments, and biological substances. ICD-10 code V: water, land, air, and space transport accidents. ICD-10 code Y: legal intervention, operations of war, military operations, and terrorism. Secondary outcomes were (a) comparison of in-hospital mortality rate for readmitted patients; (b) in-hospital mortality rate for index patients; (c) the five most common principal diagnoses for readmission; and (d) resource use associated with readmission defined as mean length of hospital stay and hospital charges. In addition, we conducted a sub-analysis assessment to determine the top causes of index hospitalizations for patients with SVI/B.

### Statistical analysis

Statistical analyses were performed using STATA, version 17.0 (Stata-Corp, College Station, TX). NRD is based on a complex sampling design, including stratification, clustering, and weighting. This software facilitates analysis to provide nationally representative unbiased results, variance estimates, and p-values. The weighting of patient-level observations was implemented to obtain estimates for the entire United States population of hospitalized patients with severe vision impairment or blindness or without. Continuous variables are presented as mean ± standard deviation (SD), and categorical variables as frequency (percentage). Proportions were compared using the chi-square, and continuous variables were compared using the Student t-test. Univariable regression model analysis was used to calculate unadjusted odds ratios for secondary outcomes. Subsequently, multivariable regression model analysis adjusted the results for potential confounders. Multivariable regression models were built by including all confounders significantly associated with the outcome on univariable analysis with a cutoff p-value of 0.1. We chose this selection method for our multivariable models because of its use in previous HCUP analyses [10]. The confounders included gender, age, hospital teaching status and location, insurance, median household income, and comorbidities measured using the Charlson comorbidity index. All p-values were 2-sided, with α <= 0.05 as a threshold for statistical significance. Survival analysis was performed with time from readmission and death as failure. The proportional hazards assumption was tested using the Schoenfeld residuals. Estimated hazard ratios (HRs) or odds ratios (ORs) are presented with 95% confidence intervals (CIs).

## Results

**Fig. 1:**
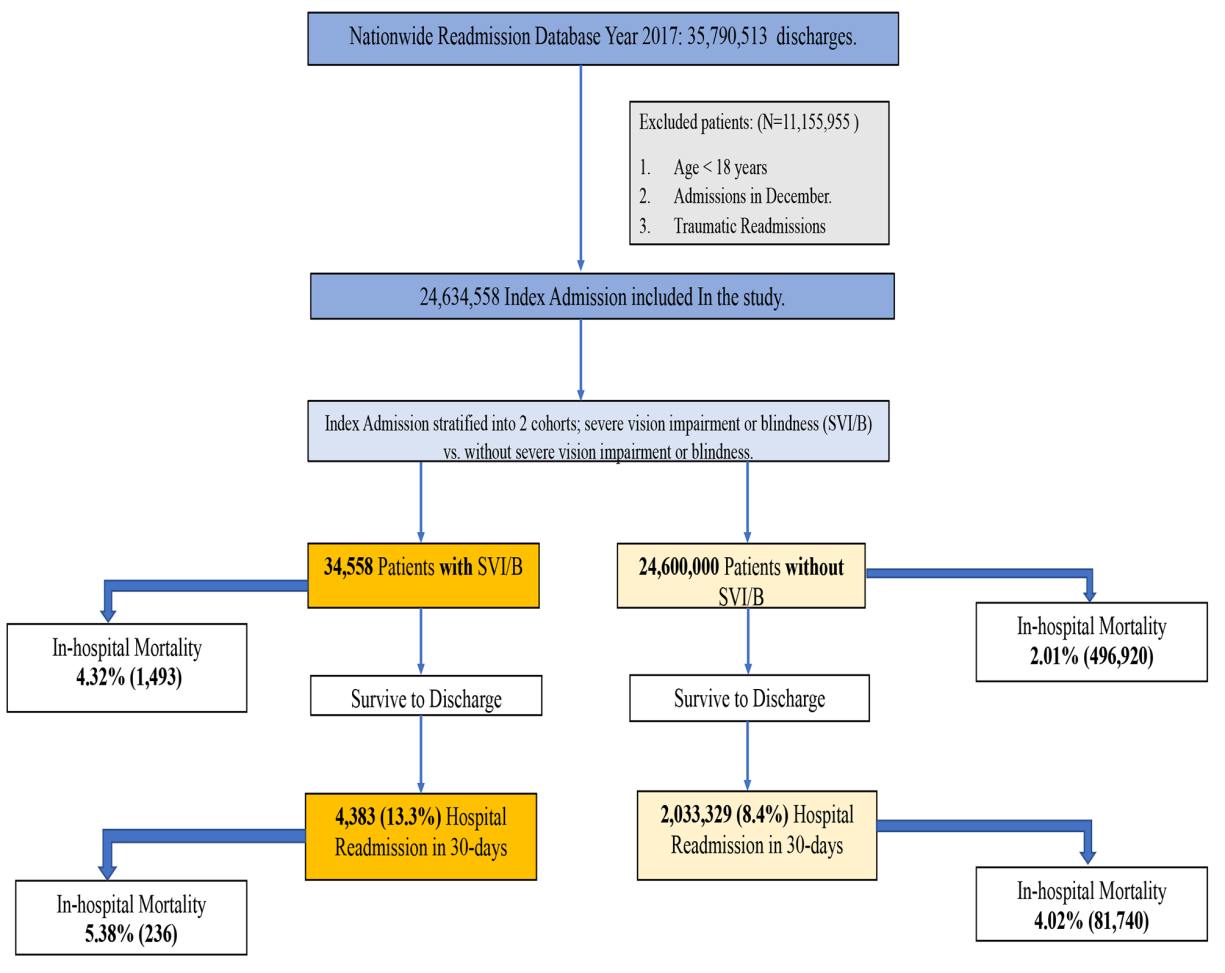
Flow diagram for our study population and outcomes for mortality and readmission

**Fig. 2:**
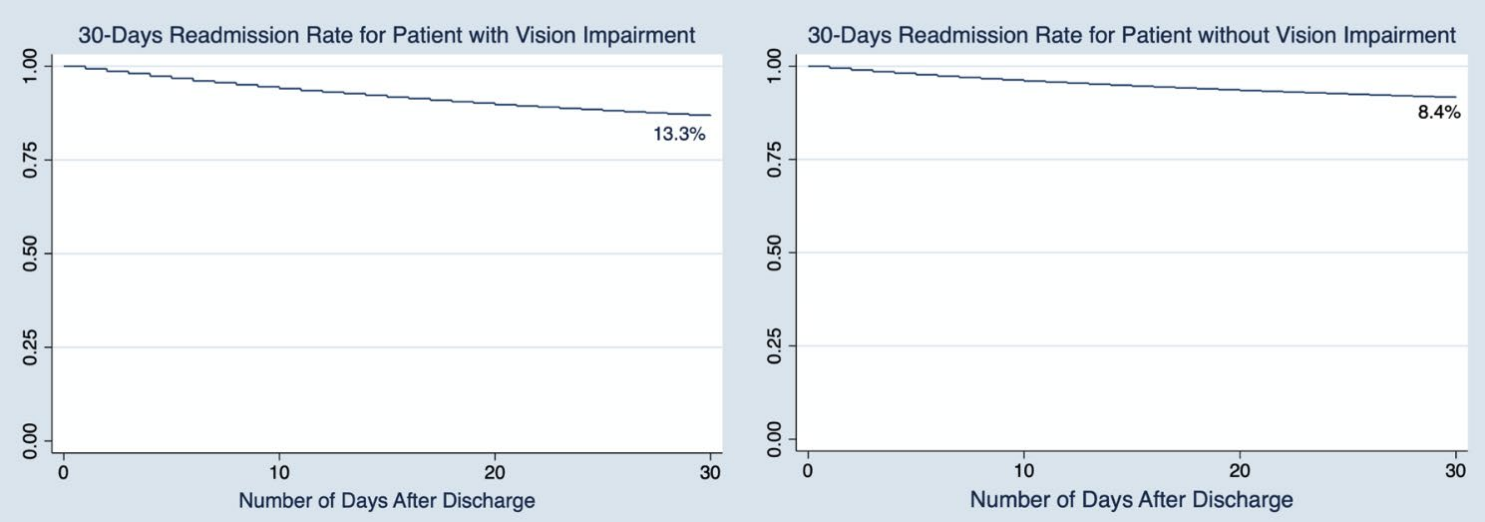
; Kaplan-Meier curve for 30-days all-cause readmission among patients with and without Severe Visual Impairment/Blindness

Figure 1 shows the flow diagram for study inclusion. From the 35,790,513 hospitalizations in 2017 in the United States, 34,558 index hospitalization (first hospitalization) patients were severely visually impaired or blind compared to 24,600,000 who were not; Patient and hospital demographic data are shown in Table 1. The 30-day rate of readmission among patients with SVI/B was 13.3% [4,383] compared to 8.4% [2,033,329] without SVI/B (Fig. 2). Among SVI/B readmitted patients, the in-hospital mortality rate was 5.38% [236] compared to 4.02% [81,740] (adjusted odds Ratio (aOR) = 1.2, 95% CI: 1.1–1.4]; p < 0.01) in those without SVI/B (Table 2). The most common reasons for readmissions among patients with SVI/B included sepsis 12% [526], heart failure 10.5% [460)], acute renal failure 4.4% [193], complications due to type II diabetes mellitus 4.1% [178], and pneumonia 2.7% [118]. Our sub-analysis revealed that the most common reasons for index admission among patients with SVI/B were sepsis 18.94% [6,545], urinary tract infection 12.44% [4,299)], hypertensive heart disease with CKD 12.36% [4,271], Pneumonia 12.14% [4,194], NSTEMI 1.78% [615] and Cerebral Infarct 1.54% [529] as represented in Table 3.

**Table 1:** Patient and hospital characteristics/demographics among readmitted patients comparing those with and without severe vision impairment or blindness: National Readmission Database (2017)

Variables	Readmitted, SVI/BI	Readmitted, No SVI/BI	p-value
	(N = 4,383)	(N = 2,033,329)	
Mean Age, in years	64.4 [SD ± 19]	61.4 [SD ± 20]	0.001
Gender			0.003
Male	2,174 (49.6%)	959,731 (47.2%)	
Female	2,209 (50.4%)	1,073,598 (52.9%)	
Insurance			< 0.001
Medicare	3,401 (77.6%)	1,187,464 (58.4%)	
Medicaid	635 (14.5%)	374,133 (18.4%)	
Private insurance	232 (5.3%)	353,833 (17.5%)	
Self-pay	52 (1.2%)	61,000 (3.0%)	
Other/Uninsured	63 (1.4%)	55,109 (2.7%)	
Hospital bed size			0.003
Small	600 (13.7%)	335,500 (16.5%)	
Medium	1,232 (28.1%)	575,431 (28.3%)	
Large	2,551 (58.2%)	1,122,398 (55.2%)	
Hospital teaching status			0.080
Metropolitan Non-Teaching	1,038 (23.7%)	471,732 (23.2%)	
Metropolitan Teaching	3,051 (69.6%)	1,374,531 (67.6%)	
Non-Metropolitan	294 (6.7%)	187,066 (9.2%)	
Median annual income expected for patient’s zip code, in US$#			< 0.001
$1 - $45,999	1,832 (41.8%)	701, 501 (34.5%)	
$46,000 - $58,999	1,118 (25.5%)	563,234 (27.7%)	
$59,000 - $78,999	855 (19.5%)	447,332 (22.0%)	
>= $79,000	578 (13.2%)	321,266 (15.8%)	
Comorbidities			
Coronary Artery Disease	662 (15.1%)	270,433 (13.3%)	0.007
Old Myocardial Infarct	460 (10.5%)	146,400 (7.2%)	0.001
Chronic Kidney Disease	2,095 (47.8%)	504,266 (24.8%)	< 0.001
Hypertension	1,227 (28%)	652,699 (32.1%)	0.001
Dyslipidemia	1,832 (41.8%)	744,198 (36.6%)	0.004
Diabetes	2,555 (58.3%)	685,232 (33.7%)	< 0.001
Chronic Liver Disease	193 (4.4%)	138,266 (6.8%)	0.090
Peripheral Vascular Disease	254 (5.8%)	83,366 (4.1%)	0.005
Congestive Heart Failure	1,398 (31.9%)	496,132 (24.4%)	< 0.001
Previous PCI	35 (0.8%)	14,233 (0.7%)	0.900
Previous CABG	272 (6.2%)	124,033 (6.1%)	0.600
Anemia	2,082 (47.5%)	648,632 (31.9%)	< 0.001
Old stroke	61 (1.4%)	16,267 (0.8%)	0.001
Obesity	548 (12.5%)	315,166 (15.5%)	0.040
Nicotine Use	964 (22%)	498,166 (24.5%)	0.009
Atrial Fibrillation	75 (1.7%)	50,833 (2.5%)	0.001
Electrolyte Abnormalities	1,683 (38.4%)	601,865 (29.6%)	0.001
Dialysis Dependent	38,633 (1.9%)	91,500 (4.5%)	0.001
COPD	802 (18.3%)	418,866 (20.6%)	0.004
Oxygen Dependent	237 (5.4%)	111,833 (5.5%)	0.900
Hypothyroidism	758 (17.3%)	272,466 (13.4%)	< 0.001
Alcohol abuse	105 (2.4%)	122,000 (6.0%)	0.010
Illicit drug use	175 (4.0%)	138,266 (6.8%)	0.030
Charlson Comorbidity Index			< 0.001
1	412 (9.4%)	437,169 (21.5%)	
2	500 (11.4%)	357,866 (17.6%)	
>=3	3,471 (79.2%)	1,238,299 (60.9%)	

Analyses used Pearson’s χ2 test and one-way analysis of variance for categorical and continuous variables, respectively.

SVI/B: Severe Vision Impairment/Blindness, PCI: Percutaneous Coronary Intervention, CABG: Coronary Artery Bypass Graft, COPD: Chronic Obstructive Pulmonary Disease.

**Table 2:** shows odd ratios and mean differences for readmission in-hospital outcomes in patients with and without severe vision impairment/blindness (SVI/B)

Outcomes	Readmitted w SVI/B (N = 4,383)	Readmitted wo SVI/B (N = 2,033,329)	aOR/aMD*	95% CI	p-value
Readmission In-hospital Mortality	5.38% (236)	4.02% (81,740)	1.2	1.1–1.4	0.016
Readmission Mean Length of Stay	6.3 days	5.3 days	0.5*	0.4–0.6	0.003
Readmission Mean Patient Charges (USD_	$57,202	$51,582	$1,379*	$1,048-$1,678	< 0.001

aOR = adjusted odd ratio, aMD = adjusted mean difference, CI = confidence interval.

Variables adjusted for age, gender, hospital teaching status, hospital location, insurance, median household income, and comorbidities were measured using the Charlson comorbidity index.

**Table 3:** Top Seven Index Admission Diagnoses Among Patients With SVI/B

Index admitting diagnosis	Number, (%)
Sepsis	6,545 (18.94%)
UTI	4,299 (12.44%)
Hypertensive heart disease with CKD	4,271 (12.36%)
Pneumonia	4,194 (12.14%)
NSTEMI	615 (1.78%)
Cerebral Infarct	529 (1.54%)
Diabetes with complication	487 (1.41%)

### Resources use during readmission compared to index admission

The mean length of stay for readmitted patients with SVI/B was 6.3 days (CI: 6.0-6.7 days), which was higher than those without SVI/B 5.6 days (CI: 5.5–5.8 days), p-value < 0.01, adjusted mean difference [aMD]: 0.5 (CI: 0.4–0.6), 95% adjusted Incidence rate ratio [aIRR] 1.18 (CI: 1.17–1.2). The mean differences in hospital charges for readmitted patients with SVI/B was $57,202 (CI: $53,712–$61,292) vs. $51,582 (CI: $49,966–$53,198), p-value < 0.001, aMD $1,379 ($1,048-$1,678), aIRR; 1.16, 95% CI: 1.11–1.23) for patients without SVI/B. Table 2 details resource utilization outcome data.

## Discussion

In the United States, patients with SVI/B had higher readmission rates, greater inpatient healthcare expenditures, and increased mortality rates upon rehospitalization than those without SVI/B. Our findings highlight the period between hospital discharge and readmissions as one of the most critical times requiring intervention to improve mortality outcomes and curtail healthcare expenditure in this vulnerable patient population. We identified the top readmitting diagnoses for patients with SVI/B, which may be targeted following an initial discharge to help curb or prevent readmissions, associated resource use, and mortality.

This work builds on previous research that SVI/B is associated with readmissions and higher healthcare costs in older patients [4]. Morse et al. studied two databases, the Medicare database, and the Clinformatics DataMart, to study readmissions among hospitalized adults between the years 2015 to 2018. The study used ICD-9 codes to study adults aged 21 years and up and found that older adults with SVI/B had greater hospitalizations, readmission, and costs. Our study for the year 2017 solely focused on using ICD-10 codes, and we also observed greater readmission and healthcare expenditure in patients with SVI/B. Because ICD-10 codes were utilized in 2017 and are more specific than ICD-9 codes, we believe our study captured more targeted patients, including younger ones. For example, patients with vision impairment studied by Morse were in their 70 years of age, while the average age for patients with SVI/B in our study was in the 60s. This is similar to past work using the National Inpatient Sample (NIS) and ICD-10 codes with patients’ mean age in the 60s [5]. However, we also captured mortality, which Morse did not study, and we observed greater mortality in those with SVI/B.

A large database study in Korea found that patients with blindness had higher mortality than those without blindness, irrespective of patient age [11]. However, an Australian study discovered no differences in inpatient mortality for hospitalized patients with blindness after adjusting for confounders [12]. Though previous work showed increased mortality among patients with SVI/B compared to those without SVI/B in the United States [5] using the National Inpatient Sample database, that study’s detection of readmissions was not possible. Our findings differed from these three past studies in that they uniquely identified readmissions among patients with SVI/B to determine mortality occurrence on index admission and upon readmission.

Given that patients with SVI/B were re-hospitalized more often than those without SVI/B, risk reduction strategies could decrease associated higher hospital expenditure, longer hospital stays, and greater mortality, all of which were observed in our study. Furthermore, understanding the needs of hospitalized patients with SVI/B could improve their safety and reduce harmful events. For example, Carlson found that visually impaired patients welcomed having bracelets to alert hospital staff [6]. Alert bracelets may lead to rapid assessments should acute medical issues arise. Thus, these bracelets may reduce in-hospital falls for patients with impaired vision.

Increasing hospital healthcare teams’ education and awareness about readmission risks and high healthcare costs for patients with SVI/B is essential to improve deleterious outcomes. Moreover, identifying the in-hospital and post-discharge care needs of patients with SVI/blindness could reduce mortality and improve patient satisfaction. Further, close outpatient follow-up with patients who have SVI/B after discharge may help identify and address risk factors that lead to rehospitalizations. It is essential to ensure that these high-risk patients can take and administer medications correctly and ask questions to clarify the discharge plan and address any issues that might have been missed before discharge [13]. It is critical that patients with SVI/B have home support needed to ensure they can comply with discharge recommendations. Without this, they are at high risk for missed medication and readmission. Like the general population, we found that patients with SVI/B are mainly admitted with sepsis, followed by heart failure [14]. Given higher readmission rates for patients with SVI/B compared to those without, aggressive interventions targeting sepsis prevention and optimal heart failure, and diabetes management post-discharge is essential to improving readmission disparities. Moreover, risk factors for readmissions due to acute renal failure and pneumonia should be further explored in this vulnerable patient population.

Several limitations of this study should be considered. First, the NRD is an administrative database wherein data is highly dependent on coding imputations. Second, the NRD lacks detailed information about visual testing results, lab data or imaging reports, and medications. Thus, an in-depth investigation into the details of our findings was not feasible. Third, special circumstances that might have influenced diagnostic or treatment decisions, such as social factors and patients’ preferences, cannot be determined from administrative databases. Fourth, disease severity and measurements documenting clinical status (improvement/worsening) over the hospitalization are not captured in NRD. Lastly, in observational studies, unmeasured and unknown confounders may influence outcomes. Observed associations suggest relationships between variables but do not prove causality.

## Conclusion

Patients with severe vision impairment or blindness are more likely to be readmitted and have higher mortality on readmission than those without vision loss. Further research is needed to determine the causes of these poor outcomes and to design effective interventions to improve care in this vulnerable patient population.

### Electronic supplementary material

Below is the link to the electronic supplementary material.

Supplementary Material 1

## Data Availability

The datasets generated and analyzed during the current study are available online in the Healthcare Cost and Utilization Project National Data Registry. This Data Use Agreement (“Agreement”) governs the disclosure and use of data in the HCUP Nationwide Databases from the Healthcare Cost and Utilization Project (HCUP), which the Agency maintains for Healthcare Research and Quality (AHRQ). Accordingly, HCUP Databases may only be released in “limited data set” form, as the Privacy Rule defines that term, 45 C.F.R. § 164.514(e). In addition, AHRQ classifies HCUP data as protected health information under the HIPAA Privacy Rule, 45 C.F.R. § 160.103. The datasets generated and analyzed during the current study are not publicly available except for the corresponding author who purchased the data and signed the HCUP Data Use agreement training. Researchers should readily be able to publicly purchase the same databases we did to conduct research.

## Abbreviations

SVI/B: Severe Vision Impairment/Blindness
NRD: Nationwide Readmission Database
SEM: Standard Estimated Mean
aOR: adjusted Odds Ratio
aIRR: adjusted Incidence rate ratio
aMD: adjusted Mean Difference
CI: Confidence Interval
ICD-10-CM: International Classification of Diseases, 10th Revision, Clinical Modification
US: United States
LOS: Length of Stay

## References

[1] Varma R, Vajaranant TS, Burkemper B, Wu S, Torres M, Hsu C, Choudhury F, McKean-Cowdin R (2016). Visual impairment and blindness in adults in the United States: demographic and Geographic Variations from 2015 to 2050. JAMA Ophthalmol.

[2] Wittenborn JS, Zhang X, Feagan CW, Crouse WL, Shrestha S, Kemper AR, Hoerger TJ, Saaddine JB, Vision Cost-Effectiveness Study Group (2013). The economic burden of vision loss and eye disorders among the United States population younger than 40 years. Ophthalmology.

[3] Rein DB, Zhang P, Wirth KE, Lee PP, Hoerger TJ, McCall N, Klein R, Tielsch JM, Vijan S, Saaddine J (2006). The economic burden of major adult visual disorders in the United States. Archives of ophthalmology (Chicago Ill : 1960).

[4] Morse AR, Seiple W, Talwar N, Lee PP, Stein JD (2019). Association of Vision loss with hospital use and costs among older adults. JAMA Ophthalmol.

[5] Harris CM, Wright SM (2021). Severe vision impairment and blindness in hospitalized patients: a retrospective nationwide study. BMC Ophthalmol.

[6] Carlson C, Howe T, Pedersen C, Yoder LH (2020). Caring for visually impaired patients in the hospital: a Multidisciplinary Quality Improvement Project. Am J Nurs.

[7] Press VG, Matthiesen MI, Ranadive A, Hariprasad SM, Meltzer DO, Arora VM (2015). Insights into inpatients with poor vision: a high value proposition. J Hosp Med.

[8] Healthcare Cost and Utilization Project—HCUP A Federal-State-Industry Partnership in Health Data. HUCP Nationwide Readmission Database (NRD). 2010–2017. Accessed August 22., 2022. https://www.hcup-us.ahrq.gov/db/nation/nrd/Introduction_NRD_2010-2017.jsp#:~:text=The%202017%20NRD%20is%20constructed,percent%20of%20all%20U.S.%20hospitalizations.

[9] Buckholtz R. Looking at new ICD-10-CM Codes for Blindness. https://www.icd10monitor.com/looking-at-new-icd-10-cm-codes-for-blindness (updated September 27th 2017) (13). Accessed August 22, 2022. Healthcare.

[10] Cecatto SB, Monteiro-Soares M, Henriques T, Monteiro E, Moura CI (2015). Derivation of a clinical decision rule for predictive factors for the development of pharyngocutaneous fistula postlaryngectomy. Braz J Otorhinolaryngol.

[11] Choi HG, Lee MJ, Lee SM (2020). Mortality and causes of death in a population with blindness in Korea: a longitudinal follow-up study using a national sample cohort. Sci Rep.

[12] Crewe JM, Spilsbury K, Morlet N, Morgan WH, Mukhtar A, Clark A, Semmens JB (2015). Health Service Use and Mortality of the Elderly Blind. Ophthalmology.

[13] Iezzoni LI. (2005, December 1). *Discharged blindly*. Patient Safety Network. Retrieved April 10, 2022, from https://psnet.ahrq.gov/web-mm/discharged-blindly.

[14] Data use agreement for HCUP Fast Stats. Most Common Diagnoses in Hospital Inpatient Stays - HCUP Fast Stats. (n.d.). Retrieved April 10., 2022, from https://www.hcup-us.ahrq.gov/faststats/NationalDiagnosesServlet.

