# Supplementary material for: Re-hospitalizations within 30-days and mortality outcomes among severely visually impaired and blind patients: analysis of the National Readmission Database
Source: BMC Ophthalmol. 2023 Aug 8;23:348. doi: 10.1186/s12886-023-03051-8 (PMC10408058; doi:10.1186/s12886-023-03051-8)
Supplement: Supplementary file 1 — Supplementary Material 1 [file 12886_2023_3051_MOESM1_ESM.docx]

Supplemental Table and Figure for Severe Vision Impairment/Blindness SVI/B.

Supplementary Table 1. International Classification of Diseases, Tenth Edition, Clinical Modification (ICD-10-CM) codes Used To Identify primary, secondary diagnoses, baseline comorbidities, procedures, and In-hospital outcomes of severe vision impairment/blindness

| Diagnoses | ICD-10 codes |
| --- | --- |
| **Severe Vision Impairment/Blindness** |  |
| Blindness right eye category 3, blindness left eye category 3 | H54.0X33 |
| Blindness right eye category 3, blindness left eye category 4 | H54.0X34 |
| Blindness right eye category 3, blindness left eye category 5 | H54.0X35 |
| Blindness right eye category 4, blindness left eye category 3 | H54.0X43 |
| Blindness right eye category 4, blindness left eye category 4 | H54.0X44 |
| Blindness right eye category 4, blindness left eye category 5 | H54.0X45 |
| Blindness right eye category 5, blindness left eye category 3 | H54.0X53 |
| Blindness right eye category 5, blindness left eye category 4 | H54.0X54 |
| Blindness right eye category 5, blindness left eye category 5 | H54.0X55 |
| Blindness right eye category 3, low vision left eye category 2 | H54.1132 |
| Blindness right eye category 4, low vision left eye category 2 | H54.1142 |
| Blindness right eye category 5, low vision left eye category 2 | H54.1152 |
| Low vision right eye category 2, blindness left eye category 3 | H54.1223 |
| Low vision right eye category 2, blindness left eye category 4 | H54.1224 |
| Low vision right eye category 2, blindness left eye category 5 | H54.1225 |
| Low vision right eye category 2, low vision left eye category 2 | H54.2X22 |
| **Comorbidities** |  |
| Coronary artery disease | I25 |
| Dyslipidemia | E78 |
| Old or Previous MI | I252 |
| Old or Previous CABG | Z951 |
| Congestive Heart Failure | I5022, I5032, I5042. |
| Protein Energy Malnutrition | E45, E46, E42, E43, E44, E440, E441 |
| Frailty/Age related Debility | R54 |
| Chronic obstructive pulmonary disease | J41, J42, J43, J44 |
| Baseline oxygen use | Z9981 |
| Carotid artery disease | I652 |
| Old or Previous stroke | I63 |
| Hypertension | I10 |
| Atrial Fibrillation | I480, I481, I482, I4891 |
| Peripheral vascular disease | I739, I738 |
| Diabetes Mellitus Type 1&2 | E10, E11 |
| Obesity | E660, E6601, E6609, E661, E662, E668, E669, Z6831, Z684. |
| Chronic liver Disease | I50 |
| Chronic Kidney Disease | N18 |
| Dialysis Dependent | Z992 |
| Chronic liver disease | K70, K71, K72, K73, K74, K75, K76, K77 |
| Electrolyte derangement/abnormalities | E870, E871, E872, E873, E874, E875, E876 |
| Nicotine Use | Z87891, F172 |
| Anemia | D50, D51, D52, D53, D55, D56, D57, D58, D59, D60, D61, D62, D63, D64 |
| Pulmonary Hypertension | I270, I272 |
| Dementia | F01, F02, F03 |
| History of Congenital Heart Disease | Q20, Q21, Q22, Q23, Q24, Q25, Q26 |
| Abbreviations: MI: Myocardial infarction, CABG: Coronary artery bypass graft, STEMI: ST-segment elevation myocardial infarction, NSTEMI: Non ST-segment elevation myocardial infarction. | |

# Regression used for Outcomes Model

# Multivariable regression models were built by including confounders signiﬁcantly associated with outcomes on univariable analysis with a cutoff p value of 0.1.

- Forward conditional step wise entry with P < 0.10
- Variables used in initial model age, gender, Insurance status, median income, hospital region, location, teaching status and Charlson comorbidities index
- Outcome variable READMITED

1. Age
2. Gender
3. Insurance status

| 1. Median income 2. Hospital location 3. Hospital teaching status 4. Hospital region  - Charlson comorbidities measures  1. AHRQ comorbidity measure: Congestive heart failure 2. AHRQ comorbidity measure: Chronic pulmonary disease 3. AHRQ comorbidity measure: Dementia 4. AHRQ comorbidity measure: Peripheral Vascular Disease 5. AHRQ comorbidity measure: Hypertension 6. AHRQ comorbidity measure: Liver disease 7. AHRQ comorbidity measure: Lymphoma 8. AHRQ comorbidity measure: Metastatic cancer 9. AHRQ comorbidity measure: Neurological disorders 10. AHRQ comorbidity measure: Chronic kidney disease 11. AHRQ comorbidity measure: Solid tumor without metastasis 12. AHRQ comorbidity measure: Peptic ulcer disease excluding bleeding 13. AHRQ comorbidity measure: Valvular disease |
| --- |
| 1. AHRQ comorbidity measure: cardiac arrhythmias 2. AHRQ comorbidity measure: coronary artery disease 3. AHRQ comorbidity measure: HIV/AIDS 4. AHRQ comorbidity measure: Rheumatoid arthritis/collagen vascular diseases 5. AHRQ comorbidity measure: Diabetes   Supplementary Figure 1 : Map Graphic of States (U.S.A.) Participating in the Nationwide Readmission Database 2017 |

**
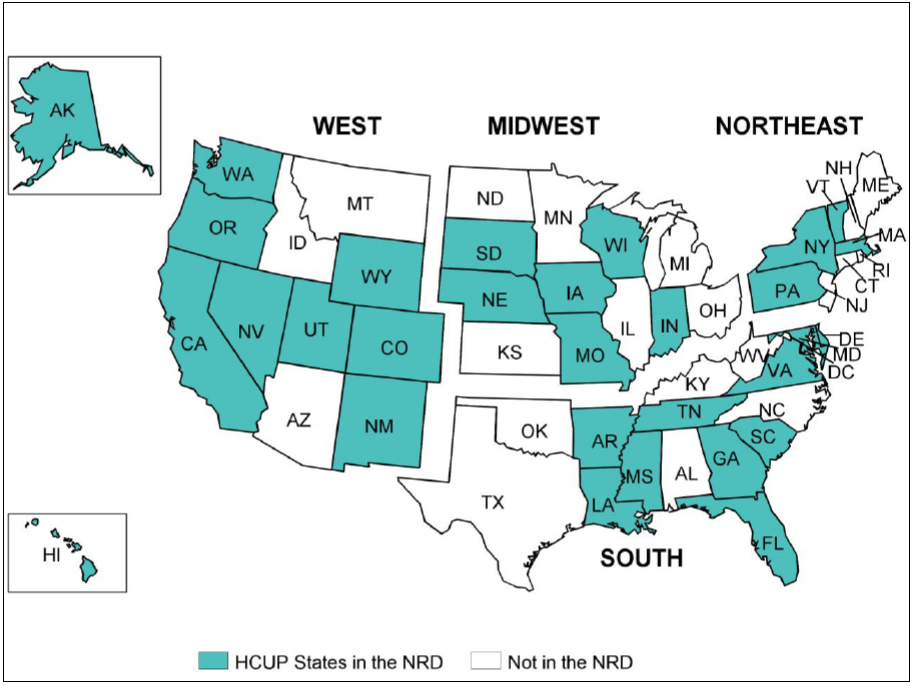
**
